# Comparative Mortality Rates of Vasoconstrictor Agents in the Management of Hepatorenal Syndrome: A Systematic Review and Meta-Analysis

**DOI:** 10.7759/cureus.67034

**Published:** 2024-08-16

**Authors:** Oluwatoba T Olayinka, Jaslin Orelus, Mah Rukh Nisar, Rudrani Kotha, Sabaa I Saad-Omer, Shivani Singh, Ann Kashmer Yu

**Affiliations:** 1 Internal Medicine, California Institute of Behavioral Neurosciences and Psychology, Fairfield, USA; 2 Emergency Medicine, California Institute of Behavioral Neurosciences and Psychology, Fairfield, USA; 3 Neurology and Medicine, California Institute of Behavioral Neurosciences and Psychology, Fairfield, USA

**Keywords:** hepatorenal syndrome, norepinephrine, noradrenaline, terlipressin, vasoconstrictor, liver failure, kidney dysfunction, hrs, aki, hepatic nephropathy

## Abstract

Hepatorenal syndrome (HRS) is an acute complication of advanced liver disease, which manifests with a rapidly progressive decline in kidney function. Though pharmacological treatment has been recently advanced, there are still high mortality rates. The study compares the mortality rate in patients using different vasoconstrictor agents in the management of HRS. A complete literature search was done in the following databases: PubMed, Cochrane Library, PubMed Central (PMC), and Multidisciplinary Digital Publishing Institute (MDPI). Studies were included according to previously established criteria, in which all studies reporting on adult patients with HRS treated with vasoconstrictor agents were eligible. The data extracted were analyzed with a random-effects model to express variability between studies, and the principal measure was the risk ratio (RR) for mortality. Of the 8,137 studies identified, 29 met the inclusion criteria. In the meta-analysis, vasoconstrictors, mainly terlipressin, significantly improved renal function and decreased the need for renal replacement therapy (RRT) versus placebo. However, a significant impact on mortality was lacking (0.94 (0.84-1.06), p = 0.31). The subgroup analysis found that mortality rates were not significantly different between vasoconstrictors, whether used in combination with or without albumin (0.97 (0.77-1.23), p = 0.79, and 0.98 (0.79-1.21), p = 0.86). Global heterogeneity was low, indicating consistent results in the studies. Vasoconstrictors are helpful in managing HRS, with improvement in renal function and reduction in RRT requirements. However, the effect on mortality was small and nonsignificant. Such findings support the use of terlipressin in HRS management; concomitantly, they emphasize the need for personalized treatment strategies and future research to find alternative therapies that may be more effective for improved survival results with fewer side effects.

## Introduction and background

Hepatorenal syndrome (HRS) is a significant complication for health conditions related to advanced liver disease. Such conditions include acute liver failure, alcoholic hepatitis, peritonitis, and cirrhosis. HRS will occur when patients with severe liver problems have a dysfunctional kidney. In 1 out of 10 patients, this leads to progressive kidney failure and, as a result, HRS [1]. While the specific cause of HRS remains unknown, the current clinical guideline is to consider liver disease patients to be at risk of HRS. Acevedo and Cramp hold that HRS can strike anyone with advanced liver disease, including people with cirrhosis of the liver and alcoholic hepatitis. Other additional risks include unstable blood pressure, gastrointestinal bleeding, spontaneous bacterial peritonitis, and large-volume paracentesis [2]. The condition is characterized by decreased urine output, azotemia (a build-up of nitrogen-containing waste products in the bloodstream), ascites, mental disorientation, jerky movements of the muscles, dark urine, nausea and vomiting, weight gain, and jaundice [1,2].

HRS is a reversible condition that is currently manageable by a myriad of therapeutic options. Treating the liver to function more efficiently and ensuring the heart can pump enough blood to the body are the two main objectives of treatment for HRS [1]. The course of treatment is the same for renal failure of any kind. In the primary standards of care, clinicians use vasoconstrictor agents and albumin. Terlipressin is one of the commonly used vasoconstrictor agents, and it is combined with albumin (a globular protein) for volume expansion [2]. Liver transplantation is considered the definitive treatment option for HRS patients. However, owing to the intricacies of securing a suitable liver to transplant, patient preferences, or underlying comorbidities, supportive measures such as renal replacement therapies (RRT) are recommended [2-4]. In further investigation of RRTs in the context of HRS, there are several commonly employed modalities to support kidney function or manage acute kidney injury (AKI). These options include continuous renal replacement therapy (CRRT), intermittent hemodialysis (IHD), peritoneal dialysis (PD), and a hybrid of CRRT and IHD or PD.

Over the last two decades, the pharmacologic treatment of HRS has evolved massively. From reports of 100% mortality rates and median two-week survival, the literature on this subject has expanded exponentially to show the effectiveness of vasoconstrictors [5]. Although recent findings have shown promising results in successfully managing HRS pharmacologically, clinicians are yet to adopt most of them into practice. At the moment, terlipressin is the sole medication with a Food and Drug Administration (FDA)-labeled indication for the treatment of HRS-AKI, according to the guidelines from the American Association for the Study of Liver Diseases in 2021 and the European Association for the Study of the Liver in 2018 [6,7]. Earlier studies, however, recommended other pharmacologic treatments for the same condition. Vasopressin analogs (ornipressin) [8], noradrenaline [9,10], alpha-adrenergic agonists (midodrine) [11], and somatostatin analogs (octreotide) [12-14] are commonly endorsed in the medical literature.

Patients with HRS have a high mortality while waiting for transplantation, and pre-transplant renal function is a primary predictor of post-transplant survival [15,16]. Research asserts that RRT is a conservative therapy in lieu of liver transplantation who are not candidates for liver transplantation. To improve kidney function and achieve better outcomes, research has demonstrated that HRS should be treated prior to liver transplantation for eligible patients [15,16]. The findings from Schoening et al.'s study [16] emphasize the importance of addressing conditions like HRS before transplantation to enhance overall patient outcomes.

The decision to use vasoconstrictor agents to manage HRS, before liver transplantation, depends on different factors, such as patients’ preference, co-morbidities, and severity of HRS [5,16]. Nevertheless, there is no clear consensus on the effectiveness of each vasoconstrictor agent. This review explores this subject to determine which modality ultimately translates to better survival outcomes in patients with HRS. The following shall be a thorough literature search of the mortality rates correlated with various vasoconstrictor agents or placebos in HRS.

## Review

Methodology

Study Design

This is a systematic review and meta-analysis conducted according to the Preferred Reporting Items for Systematic Reviews and Meta-Analyses (PRISMA) study characteristics guidelines [17].

Literature Search

An electronic search was performed on four databases (PubMed, Cochrane Library, PubMed Central (PMC), and Multidisciplinary Digital Publishing Institute (MDPI)). A supplementary search for grey literature on Google Scholar followed. The study search utilized keywords and keyword combinations for the initial search. Baseline keywords were “hepatorenal syndrome” AND “vasoconstrictor agents” AND “mortality”. Boolean operators (AND/OR), medical subject headings (MeSH) terms, truncations, and filters were applied to build search strings for each database scoured. When building the search string, the commonly used vasoconstrictor agents were used as keyword combinations for the keyword “vasoconstrictor agents”.

Eligibility Criteria

For this review, studies were selected based on predetermined eligibility criteria: All studies published until April 2024 were accepted for inclusion. Ongoing clinical trials were excluded due to a lack of complete data. A Population, Intervention, Comparator, Outcome (PICO) criteria was adopted to formulate the eligibility criteria. (1) Studies were accepted if they included adult (18+ years) patients/population (P) with HRS, hepatic nephropathy, AKI with HRS, liver-kidney syndrome, or kidney dysfunction caused by liver failure. The patient’s gender and geographical location of the study were irrelevant. (2) The primary intervention (I) of interest was the use of a vasoconstrictor agent or a combination of a vasoconstrictor agent and albumin. (3) Studies would only be accepted if the control (C) intervention was another vasoconstrictor agent, a placebo intervention, or standard care for HRS. For this criteria, the following vasoconstrictor agents were reviewed: Terlipressin, noradrenaline, norepinephrine, midodrine, octreotide, vasopressin, arginine vasopressin, dopamine, phenylephrine, epinephrine, and dobutamine. (4) The study was interested in the outcome (O) of patient survival or mortality rate following treatment with either. A secondary outcome of interest was the patient’s need for RRT or liver transplant after medication. The studies were also screened for their study designs and eligible study designs were cohort studies, case-control studies, cross-sectional studies, observational studies, randomized controlled studies (RCTs), and longitudinal retrospective studies. Only studies published in English or translated into English versions were included. The review excluded case reports, meta-analysis/systematic reviews, economic analyses, animal studies, cadaver studies, narrative reviews, or editorials.

Data Extraction

Data was extracted to a standardized Google Sheet. A 4-point data extraction was executed, focusing on the following data points. (1) Study characteristics: Author(s) of the study, year of publication, and study design; (2) Patient demographics: Number of participants, age, sex, and the presenting HRS characteristics; (3) Intervention details: Type of treatment intervention received and the comparator intervention; (4) Mortality rates and other relevant outcomes post-treatment. We also extracted the concluding comments from the study authors.

Statistical Analysis

Extracted quantitative data was analyzed on Review Manager (RevMan 5.4; The Cochrane Collaboration, Oxford, UK) using a random-effects model to accommodate expected clinical and methodological variability among the studies. This model assumes that the effect sizes vary between studies, thus providing a more generalized estimate. The primary measure of effectiveness was the risk ratio (RR), which was calculated at a 95% confidence interval (CI). The log transformation of RRs was utilized to normalize the distributions and stabilize variances.

Heterogeneity was further explored by I² statistic, which quantifies the proportion of the total variation across studies that results from heterogeneity rather than chance. The basis of judgment was that I² values between 25% and 75% were considered to represent low to high heterogeneity. Similarly, a test of the significance of heterogeneity was done with a Tau² and Chi-squared test of the same. We tested the strength of findings by sensitivity analysis, a process that involved removing one study at a time to see its impact on the overall effect. Funnel plots were used to check publication bias, and the results of the meta-analysis were summarized on a forest plot. This cautious approach helped make a conclusion that can be considered reliable regarding the comparative efficacy of vasoconstrictors in managing HRS.

Results

Study Selection

**Figure 1:**
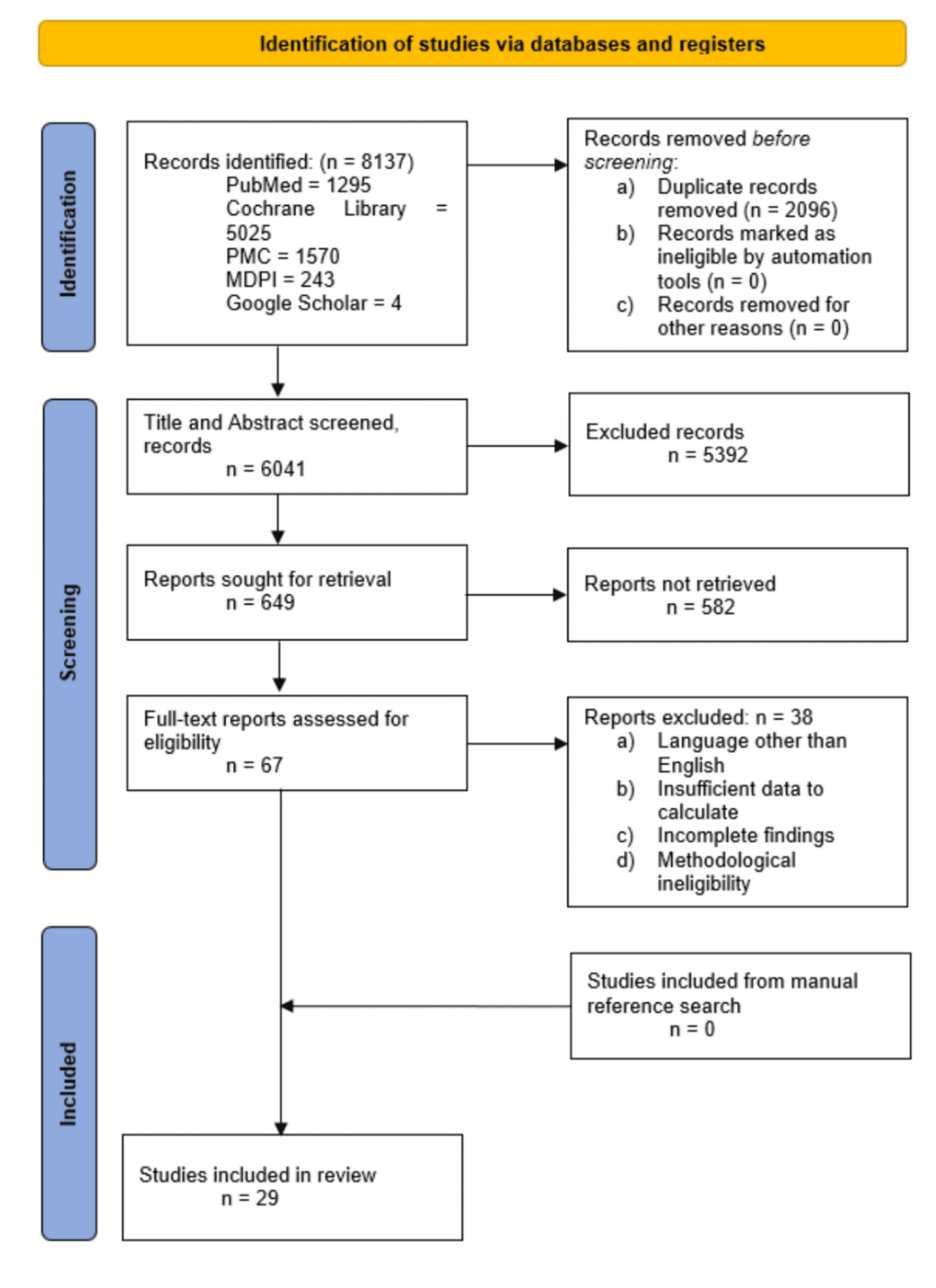
PRISMA flow diagram outlining the study selection process for studies included in the systematic review and meta-analysis. PRISMA: Preferred Reporting Items for Systematic Reviews and Meta-Analyses; PMC: PubMed Central; MDPI: Multidisciplinary Digital Publishing Institute

The search resulted in 8137 published studies on the comparative efficacy of vasoconstrictors, each following an array of thematic approaches identified. A total of 2096 studies were eliminated due to duplication and after meeting the exclusion criteria. From the remaining 6041 articles, a title and abstract screening was performed. From this stage, 5392 studies were eliminated, leaving only 649 articles. The screening process sought to retrieve all these and ended up with 67 studies. The final screening phase was a full-text screening process where three studies were eliminated. This was done for primary methodological reasons, including study designs being ineligible, outcomes that differed from what was required, and findings that were considered redundant, retracted, or otherwise incomplete. Meta-analyses, systematic reviews, study protocols, and plot studies were also eliminated. A manual reference search did not yield any new studies eligible for inclusion. Only 29 studies were included in this systematic literature review and meta-analysis. Figure 1 represents a 2020 PRISMA flowchart diagram that shows the selection process of the included studies.

Study Characteristics

Table 1 provides general details of data collected from the numerous studies that have described the results of mortality in HRS patients treated with different vasoconstrictor agents. The table consists of the necessary specifications including study design, patient information, intervention, comparators, results, and comments. Such a detailed comparison makes it possible to determine the effectiveness and safety of agents like terlipressin, noradrenaline, and other used treatments in the preservation of renal function as well as the survival rate among HRS patients. The summed data is helpful in assessing the treatment regimens and clinical decisions for HRS management.

**Table 1:** Table of data extracted from studies reporting the outcome of mortality in HRS patients after treatment with vasoconstrictor agents. HRS: Hepatorenal syndrome

Author	Study Design	Demographics	Intervention	Comparator	Comparator 2	Results	Comments
Alessandria et al., 2007 [10]	Prospective, randomized, unblinded, pilot study	Age: 56 vs. 55, Male: 7 vs. 9, Female:3 vs. 3, Type I Hepatorenal Syndrome: 4 VS. 5, Type II: 6 vs. 7, Child-Pugh score: 10 ± 1 vs. 11 ± 1	Noradrenalin and albumin: n = 10	Terlipressin and albumin: n = 12	_	Serum Creatinine (mg/dL): 1.3 ± 0.1 vs. 1.3 ± 0.2, Urine volume (mL/d): 1583 ± 243 vs. 1578 ± 314, Complete response: 7 vs. 10, Dead: 3 vs. 4	Noradrenalin is as effective and safe as terlipressin in patients with Hepatorenal Syndrome
Arora et al., 2020 [18]	Randomized trial	Age: 40.26 vs. 38.80, male: 58 vs. 55, female: 2vs. 5, Drug-induced liver injury: 4 vs. 5, Hepatitis E Virus infection: 4 vs. 2, Stage of Acute Kidney Injury: II: 31 vs. 32, III: 29 vs. 28	Terlipressin: n = 60	Noradrenaline: n = 60	_	Survival: 29 vs. 12, response day 7: 25 vs. 12	Infusion of terlipressin gives an earlier and higher response than noradrenaline, with improved survival in Acute-on-Chronic Liver Failure patients with Hepatorenal Syndrome-Acute Kidney Injury.
Boyer et al., 2016 [19]	Randomized controlled study	Age: 55.8 vs. 54.8 yrs, Female: 45 vs. 32, Male: 52 vs. 67, Child-Pugh score: 10.4 vs. 10.3, Alcoholic hepatitis: 20 vs. 25	Terlipressin: n = 97	Placebo: n = 99	_	Response: Male: 12/52 vs. 10/67, Female: 7/45 vs. 3/32, Deaths: 40 VS. 43, Multi-organ failure: 10 VS. 5, Pulmonary edema: 10 vs. 7	Terlipressin plus albumin was associated with greater improvement in renal function vs albumin alone in patients with cirrhosis and Hepatorenal Syndrome type 1.
Cavallin et al., 2015 [20]	Randomized trial	Age: 60 vs. 65 yrs, Male: 21 vs. 11, Female: 6 vs. 10, Etiology (viral/non-viral): 10/17 vs. 8/13, Hepatorenal syndrome type 1: 25 vs. 19, type 2: 2 vs. 2	Terlipressin with albumin: n = 27	Midodrine and octreotide plus albumin: n =22	_	Survival: 16 vs. 9, Serum creatinine, mmol/L (mg/dL): 3.6 vs. 3.8, Heart rate, bpm: 78.4 vs. 80.6	There was a significantly higher rate of recovery of renal function in the Terlipressin group compared to the Midodrine/Octreotide group.
Cavallin et al., 2016 [21]	Randomized trial	Age: 57.41 vs. 59.41, Male: 24 vs. 24, Female: 10 vs. 13, Etiology, viral/not viral: 15/19 vs. 16/21	Albumin + Continuous intravenous infusion of terlipressin: n = 34	Albumin + intravenous boluses of terlipressin: n = 37	_	Survival: 23 vs. 20, complete response: 19 vs. 17, partial response: 7 vs. 7, Circulatory overload: 2 vs. 5	Terlipressin given by continuous intravenous infusion is better tolerated than intravenous boluses in the treatment of type 1 Hepatorenal syndrome.
Chertow et al., 1996 [22]	Randomized, double-blind, placebo-controlled clinical trial	Age: 60.1 vs. 63.5 vs. 62.3 yrs, Male:46 vs. 63 vs. 57, Female: 33 vs. 23 vs. 34, Myocardial infarction: 7 vs. 17 vs. 14	None: n = 79	Dopamine <3 ug/kg/min: n = 86	Dopamine >3ug/kg/min: n =91	Hypertension: 25 vs. 18 vs. 28, immunosuppression: 6 vs. 3 vs. 5, Chronic obstructive pulmonary disease: 2 vs. 5 vs. 3,	There is insufficient evidence that the administration of low-dose dopamine improves survival or obviates the need for dialysis in persons with acute renal failure.
El-Desoki Mahmoud et al., 2021 [23]	Randomized controlled trial	Age: 61.85 vs. 59.92 yrs, Weight: 74.40 vs. 78.84 kg, Male: 12 vs. 18, Female: 18 vs. 12 child-pugh score: 12.13 vs. 11.37	Midodrine/octreotide + albumin: n = 30	Norepinephrine + albumin: n = 30	_	Surviving patients: 6 vs. 11, Responders:5 vs. 15 HRS reversal:11 vs. 13, Hepatic encephalopathy: 12 vs. 7	Norepinephrine plus albumin is significantly more effective than midodrine and octreotide plus albumin in improving renal function in patients
Fathallah et al., 2023 [24]	Prospective randomized controlled	Age: 53.1 vs. 52.1 yrs, Male: 11 vs. 13, Female: 9 vs. 7, Acute kidney injury: Stage I: 13 vs. 10, Stage II: 7 vs. 10	Terlipressin: n = 20	Norepinephin: n = 20	_	Serum Creatinine (mg/dL): 1.18± 0.2 vs. 1.29±0.21, Urine output (ml/24 h): 886.36±164.455 vs. 844.44±148.84, Survivors: 11 vs. 9	Norepinephrine and terlipressin had nearly similar response rates for the treatment of type 1 Hepatorenal Syndrome.
Giustino et al., 2015 [25]	Prospective, double-blinded, multicentre study	Age: 71.3 vs. 71.6 yrs, Male: 65 vs. 63, Female: 9 vs. 9, Diabetes mellitus: 30 vs. 37	Ioxaglate (n =74)	Iodixanol (n = 72)	_	30-day Deaths: 1 vs. 4, Revascularization: 1 vs. 2, Acute renal failure: 18 vs. 14, Need for dialysis: 3 vs. 1, 1-Year deaths: 3 vs. 9	The use of ionic low-osmolar contrast media ioxaglate was associated with numerically lower mortality at 1 year as compared to iodixanol in patients who underwent cardiac catheterization.
Indrabi et al., 2013 [26]	Randomized prospective study	Type 1 hepatorenal syndrome	Noradrenaline and albumin: n = 30	terlipressin and albumin: n = 30	_	Reversal of hepatorenal syndrome: 16 vs. 17, Survival at 90 days: 1 vs. 2	There is no difference in the outcome of patients of hepatorenal syndrome treated with noradrenaline or terlipressin, thus noradrenalin which is cheaper can be used instead of terlipressin
Karvellas et al., 2023 [27]	Retrospective study	Age: 54 yrs. Vs. 50 yrs, Male: 21 vs. 6, Female: 33 vs. 44, Hepatitis C: 5 vs. 4, Mean arterial pressure (mm Hg): 80 vs. 79	Terlipressin (n = 31)	Placebo (n = 14)	_	90-day Mortality: 6/31 alive Vs. 5/14 alive in placebo, Time from Intensive Care Unit admission to death (d): 9 vs. 12 days	Patients admitted to the Intensive Care Unit with Hepatorenal Syndrome-Acute Kidney Injury who received terlipressin were more likely to achieve renal function improvement, based on serum creatinine changes by the end of treatment, and had significantly shorter lengths of Intensive Care Unit stay than patients randomized to the placebo arm.
Kulkarni et al., 2022 [28]	Prospective cohort study	Age: 48.31 yrs, Male: 109, Female: 7, Etiology of liver disease: alcohol/Non-alcoholic Steatohepatitis/Hepatitis B Virus/Unknown/Hepatitis C Virus: 82/20/11/2/1	Terlipressin: n = 116	_	_	Abdominal pain: 2, Myocardial ischemia: 1, Cyanosis + arrhythmia: 1, Mortality: 49	Terlipressin non-response predicts mortality in patients with Acute-on-Chronic Liver Failure and Hepatorenal Syndrome-Acute Kidney Injury.
Martín-Llahí et al., 2008 [29]	Randomized study	Age: 59 vs. 55, Male: 16 vs. 13, Female: 7 vs. 10, Hepatorenal syndrome: 17 vs. 18, Alcoholic cirrhosis: 14 vs. 19, Hepatic encephalopathy: 10 vs. 10, Central venous pressure (cm H2O): 11 vs. 10	Terlipressin + albumin: n = 23	Albumin: n = 23	_	Circulatory overload: 7 vs. 4, Myocardial infarction: 1 vs. 0, Arrhythmia: 2 vs. 0, Circulatory overload: 7 vs. 4, Arterial hypertension: 1 vs. 0, Mortality: Those alive: 6 vs. 4	Terlipressin and albumin are more effective in improving renal function in patients with cirrhosis and hepatorenal syndrome.
Moore et al., 2020 [30]	Multicentre randomized study	Age:53.9 vs. 50.7, Male: 136 vs. 16, Hepatitis C:29 vs. 7, Alcoholic liver disease: 140 vs.12	Terlipressin (N = 203)	Other vasopressors (N = 22)	_	Complete Response: 102 vbs. 5, Overall Response: 148 vs. 13, Mortality: 55 vs. 9, Multi-organ failure: 18 vs. 2, Myocardial infarction: 2 vs. 0	Treatment with terlipressin in patients with less severe acute kidney injury (serum creatinine <2.25 mg/dL) was associated with higher treatment responses, and 90-day survival.
Nayyar et al., 2021 [31]	Prospective study	Age: 54.64 vs. 53.45, Males: 16 vs. 24, Etiology (%): Alcohol: 63.6, Hepatitis C: 27.3 vs. 44.8,	Noradrenaline: n = 22	Terlipressin: n = 22	_	Mortality: 8 vs. 1, urine output change: 287.86 +/- 93.99 vs. 860.52+/- 102.18 mL/day, arterial pressure change: 12.55 vs. 14.37 mmHg, serum creatinine change: 0.84 vs. 0.28 mg/dL	Noradrenaline was not as effective as terlipressin.
Nowsherwan et al., 2021 [32]	Randomized controlled trial	Age: 40.64 vs. 42.55, Mean BMI (24.2 vs. 23.61, Male: 21 vs. 23, Female: 27 vs. 25	Terlipressin + albumin: n = 48	Albumin: n =48	_	Mortality: 9 vs. 24	Hepatorenal syndrome Terlipressine was more effective than albumin only.
Ortega et al., 2002 [33]	Prospective, nonrandomized study	Age: 56.2 vs. 60.3, Male: 7 vs. 7, Female: 6 vs. 1, Etiology: alcohol: 3 vs 1, Child-Pugh score: 11 vs. 10, serum creatinine: 3.6 vs. 3.4 mg/dL	Terlipressin + albumin: n = 13	Terlipressin: n = 8	_	Mortality: 5 vs. 6, Mean arterial pressure (mm Hg): 79 vs. 60, Urine volume (mL/d): 1,057 vs. 739	Albumin appears to improve markedly the beneficial effects of Terlipressin.
Saif et al., 2018 [34]	Randomized controlled trial	Age: 51.5 vs. 53.8 yrs, Child-Pugh score: 12 vs. 11.9, Serum sodium (mEq/L): 119.4 vs. 118.5	Noradrenaline: n = 30	Terlipressin: n = 30	_	Survival: 8 vs. 13, Urine output at the end of treatment (mL/day): 807.1 ± 504.1 vs. 1037.5 ± 472.1, Creatinine at the end of treatment (mg/dL): 2.3 ± 1.5 vs. 1.6 ± 1.1	There is no difference in the outcome of patients from either of the two.
Sanyal et al., 2008 [35]	Randomized, prospective, double-blind, placebo-controlled trial	Age: 50.6 vs. 52.9, Male: 41 vs. 39, Female: 15 vs. 17, Child-Pugh score: 11.7 (1.9) vs. 11.2 (1.8), Hepatitis C: 22 vs. 19, Hepatitis B: 4 vs. 1	Terlipressin: n = 56	Placebo: n = 56	_	Hepatorenal syndrome reversal: 19 vs. 7, survival vs. 21, Serious Adverse Events up to 30 days post-treatment: 37 vs. 36	Terlipressin is an effective treatment to improve renal function in Hepatorenal syndrome type 1.
Sanyal et al., 2017 [36]	Reverse randomized clinical studies	Age: 53.9 vs. 54.2, Male: 93 vs. 106, Female: 60 vs. 49, Alcoholic hepatitis: 40 VS. 45, Encephalopathy stage: 1.5 vs. 1.4	Terlipressin n = 153	Placebo n = 155	_	Survival: 83 vs. 77, Mean arterial pressure: +4.1 mmHg vs. - 1.8 mmHg, HRS reversal: 42 vs. 22	Terlipressin plus albumin resulted in a significantly higher rate of Hepatorenal syndrome reversal vs. albumin alone in patients with Hepatorenal syndrome type 1.
Singh et al., 2012 [37]	Randomized control trial	Age: 51.4 vs. 48.3 yrs, Male: 10 vs. 19, Female: 4 vs. 4, Etiology: Alcohol: 10 vs. 12, Others: 13 vs. 11 Child-Turcotte-Pugh score: 10.70 vs. 10.43	Terlipressin n = 23	Noradrenaline: n = 23	_	Responders: 9 vs. 10, Urine output (ml/d): 1084 ± 417 vs. 1393 ± 529, Mean arterial pressure (mmHg): 70.6 ± 11.2 vs. 80.3 ± 5.9, Survival: 7 vs. 8	Noradrenaline is as safe and effective as terlipressin, but less expensive in the treatment of Hepatorenal syndrome and baseline Child-Turcotte-Pugh score is predictive of response.
Solanki et al., 2003 [38]	Randomized controlled single-blind trial	Age: 51 vs. 52 yrs, Male: 9 vs. 8, Female: 3 vs. 4, Hepatic encephalopathy grade I: 9 vs. 10, II-IV: 3 vs. 2	Terlipressin n = 12	Placebo: 12	_	Survival: 5 vs. 0, Urine output (mL/24 h): 1068 ± 56 vs. 291 ± 45, Mean arterial pressure (mmHg): 95 ± 1.6 vs. 70 ± 1.4	In patients with Hepatorenal syndrome, terlipressin significantly improved renal functions and systemic hemodynamics, and showed a trend towards better clinical outcomes.
Srivastava et al., 2015a -(HRS I) [39]	Open-label, randomized trial	Age: 45.8 vs. 39.2, Male: 19 vs. 17, Female: 1 vs. 3, Etiology: Alcohol: 20 vs. 0, HBV: 6 VS. 0, HCV: 3 VS. 0	Terlipressin n = 20	Triple therapy: n = 20	_	Survival: 3 vs. 3, Urine output (ml/day: 765.0 699.27 vs. 706.5 595.45, Serum creatinine (mg/dl): 3.6 1.83 vs. 3.7 2.25	Concurrent triple therapy improved renal function in Hepatorenal syndrome and was less expensive than terlipressin.
Srivastava et al., 2015b - (HRS II) [39]	Open-label, randomized trial	Age: 44.6 vs. 42.6, Male: 17 vs. 16, Etiology: Alcohol: 21 vs. 0, HBV: 7 VS. 0, HCV: 4 VS. 0	Terlipressin n = 20	Triple therapy: n = 20	_	Survival: 6 vs. 5, Urine output (ml/day: 1247.5 921.59 vs. 1139.5 627.02, Serum creatinine (mg/dl): 1.6 0.58 vs. 1.5 0.71	Concurrent triple therapy improved renal function in Hepatorenal syndrome and was less expensive than terlipressin.
Ullah et al., 2022 [40]	Randomized prospective study	Age: 40.26 vs. 41.81, Male: 27 vs. 26, Female: 13 vs. 14, Hepatitis C: 30 vs. 31, Hepatitis B: 13 vs. 10, Hepatic encephalopathy: 13 vs. 9	Terlipressin with albumin: n = 40	Norepinephrine and albumin: n = 40	_	Survival: 21 vs. 19, Serum creatinine (mg/day): 1.26±0.65 vs. 1.21±0.39, Urine output (ml/24 h): 1148.04±196.95 vs. 1192.13±136.61	Norepinephrine in combination with albumin is as effective as terlipressin in combination with albumin when used for the management of hepatorenal syndrome (HRS) type 1.
Velez et al., 2023 [41]	Retrospective study	Age: 54 vs. 54, Male: 213 vs. 165, Female: 139 vs. 91, Etiology: Alcohol: 212 vs. 150, Hepatitis B: 11 vs. 5, Hepatitis C: 90 vs. 68	Terlipressin n = 352	Placebo: n = 256	_	Survival: 130 vs. 73, need for renal replacement therapy (RRT): 106 vs. 97, need for post-liver transplantation (LT): 72 vs. 103	Terlipressin decreased the requirement of Renal Replacement Therapy compared with placebo among patients with Hepatorenal syndrome type-1.
Wong et al., 2017 [42]	Retrospective study	Age: 56.3 vs. 54.1, Male: 13 vs. 22, Female: 15 vs. 8, Alcoholic hepatitis: 7 vs. 12, Mean arterial pressure (MAP) (mm Hg):74.6 (± 11.5) vs. 76.4 (± 10.8)	Terlipressin n = 28	Placebo: n = 30	_	HRS reversal: 12 vs. 2, Survival: 13 vs. 7, MAP (mm Hg): 83.1 (± 13.77) vs. 74.2 (± 12.06)	Terlipressin improved renal function and reversed Hepatorenal syndrome in a higher proportion of patients with Hepatorenal syndrome type 1 and Systemic Inflammatory Response Syndrome (SIRS) than patients who received albumin plus placebo.
Wong et al., 2021 [43]	Randomized, double-blind, placebo-controlled trial	Age: 54 vs. 53.6, Male: 120 vs.59, Female: 79 vs. 42, Alcoholic hepatitis: 81 vs. 39, Mean arterial pressure - mm Hg: 78.7±12.1 vs. 77.5±9.4, Etiology: alcohol: 134 vs. 67, Nonalcoholic steatohepatitis: 42 vs. 24, Viral hepatitis: 35 vs. 8, Autoimmune hepatitis: 10 vs. 5	Terlipressin n = 199	Placebo: n = 101	_	HRS reversal: 63 vs. 17, Death: 101 vs. 45, Cardiac disorders: 8 vs. 6, Cardiac disorders: 1 vs.3, Gastrointestinal hemorrhage: 8 vs. 0	Terlipressin was more effective than placebo in improving renal function but was associated with serious adverse events, including respiratory failure.
Wong et al., 2022 [44]		Age: 55.7 vs. 54.6, Male: 94 vs. 48, Female: 66 vs. 33, Etiology: Alcohol: 101 vs. 52, Viral hepatitis: 28 vs. 4, Primary biliary Cholangitis: 4 vs. 3, Auto-immune: 6 vs. 3	Terlipressin: n = 160	Placebo: n = 81	_	Death: 101 vs. 45, Respiratory failure (RF): 16 vs. 3	Terlipressin should be used with caution in patients with Hepatorenal syndrome type 1 and grade 3 Acute-on-Chronic Liver Failure. Patients with hypoxemia are at increased risk of respiratory failure and mortality.

Meta-Analysis

**Figure 2:**
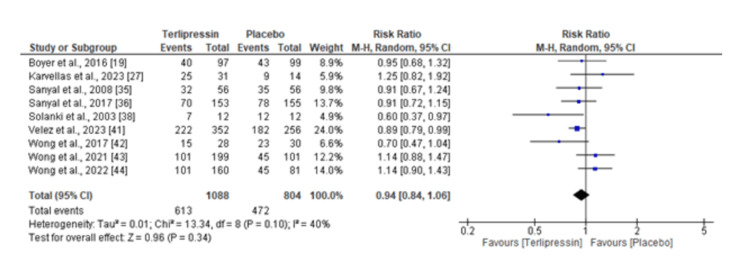
Forest plot of meta-analysis results calculating the relative morality risk of vasoconstrictors compared to placebo.

Vasoconstrictors (terlipressin) vs. placebo: For this analysis, the relative mortality risks of vasoconstrictors (with a keen focus on terlipressin) compared to placebo in treating HRS were assessed. This was a significant analysis, including multiple studies contributing to the overall RR of 0.94, 95% CI: 0.84-1.06. This suggests that terlipressin has a small (non-statistically significant) reduction (6%) in the risk of mortality and has no difference in effectiveness when compared to placebo. The analysis had moderate heterogeneity (I² = 40%), which means that the studies are different, and the differences are observed in the dispersion of effects between studies. For instance, Velez et al. [41] and Sanyal et al. [35] show more favorable results for terlipressin. That is because the CI is narrower and more accurate. On the other hand, studies such as Karvellas et al. [27] have wider intervals, hence, are less precise and have mixed results that sometimes favor the placebo. The forest plot in Figure 2 presents the findings of this analysis.

**Figure 3:**
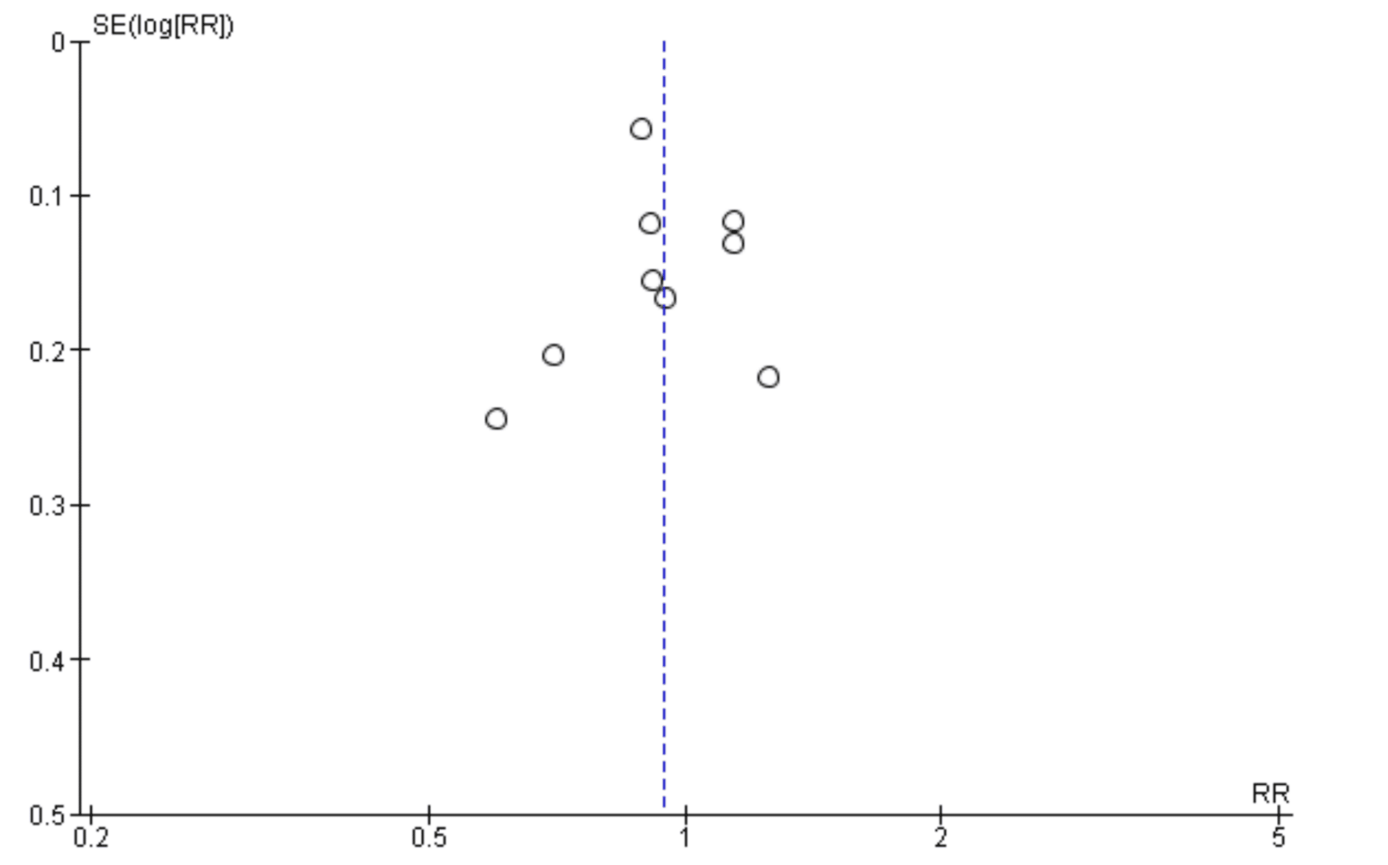
Funnel plot showing the level of publication bias between studies analyzing mortality rates. RR: risk ratio

Assessing the publication bias of the included studies, we find that the spread of studies around the vertical line shows the effect size of RR = 1, which indicates symmetry and hence no/minimal publication bias. The vertical distribution against standard error (SE) shows that smaller studies have higher SE. In comparison, larger studies have lower SE, and thus, both are evenly distributed around the summary effect size, indicating reliability and lack of bias in this meta-analysis. The funnel plot in Figure 3 presents the findings on publication bias and heterogeneity in meta-analyses.

**Figure 4:**
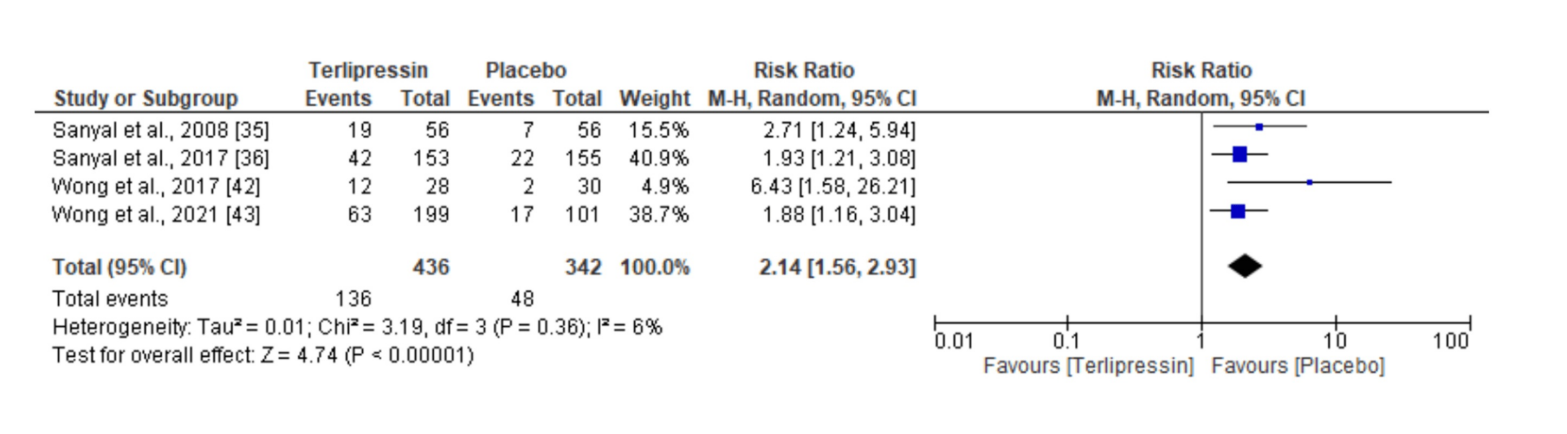
Forest plot of meta-analysis results calculating the rates of HRS reversal between vasoconstrictors compared to placebo. HRS: Hepatorenal syndrome

The results suggest a more than double likelihood of reversing HRS by vasoconstrictors (terlipressin) compared with placebo, with a pooled RR of 2.14, 95% CI 1.56 to 2.93. This estimate was statistically significant, suggesting a strong therapeutic potential. The studies by Sanyal et al. [36] and Wong et al. [43], which account for 40.9% and 38.7% of the analysis weight, report high values of RR and hence contribute to terlipressin being strongly efficacious. However, the overall heterogeneity between the studies is low, at 6%, which indicates that among the studies, there are consistent results but with very little variability due to sampling error but no true differences in study outcomes. Figure 4 presents the findings of this analysis in a forest plot.

**Figure 5:**
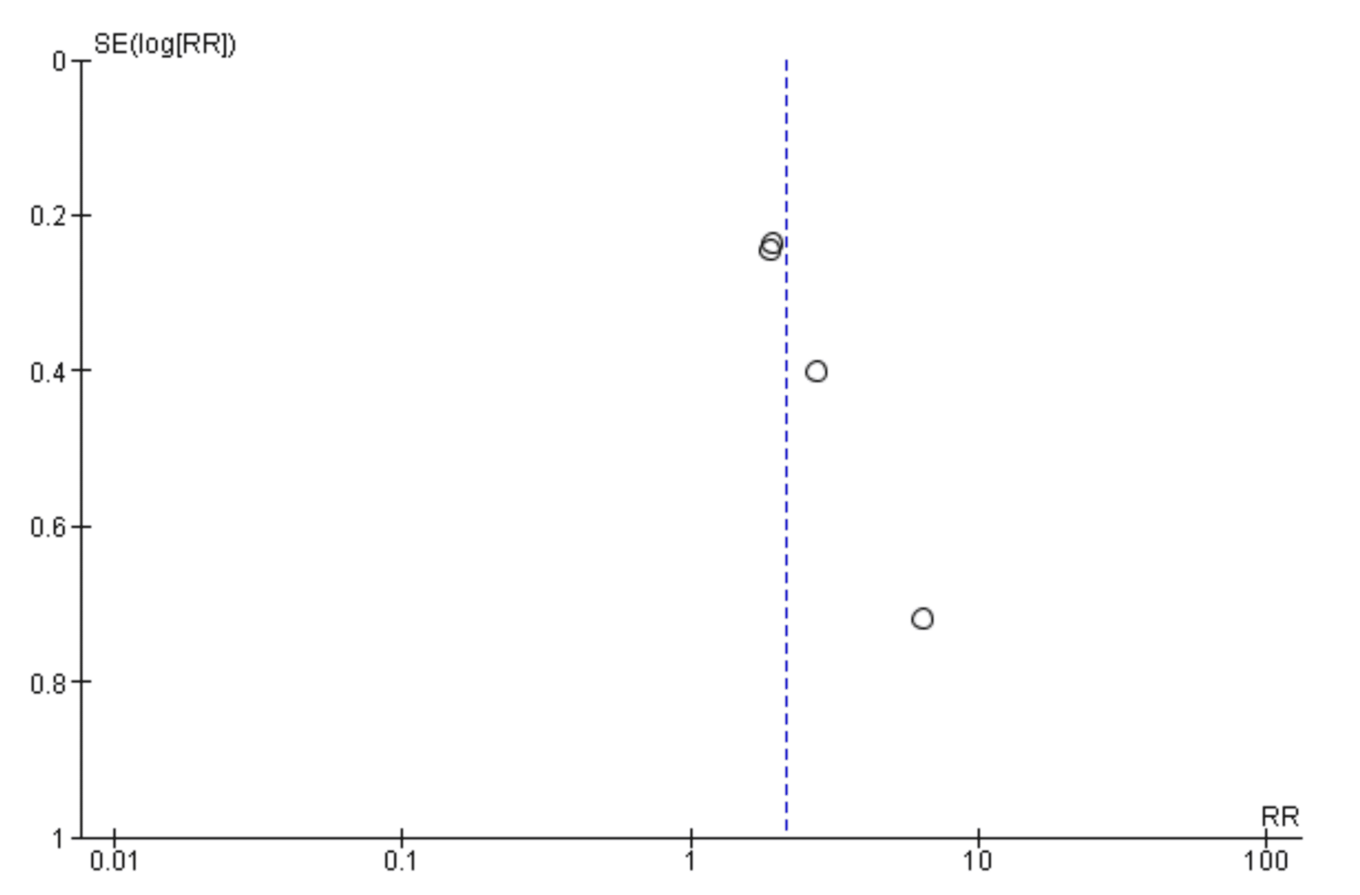
Funnel plot showing the level of publication bias between studies analyzing rates of HRS reversal. RR: risk ratio; HRS: Hepatorenal syndrome

An assessment of publication bias was necessary to confirm the validity of the results. Figure 5, which features a funnel plot, assessed publication bias and heterogeneity in this outcome analysis. Most of the studies are clustered around the top, close to the line of no effect (RR = 1), so the spread is small, and there is underrepresentation of smaller or negative studies. There is one isolated study at the bottom of this funnel that would denote a potential outlier or higher variability in smaller studies, leading to bias in generalizing the results.

**Figure 6:**
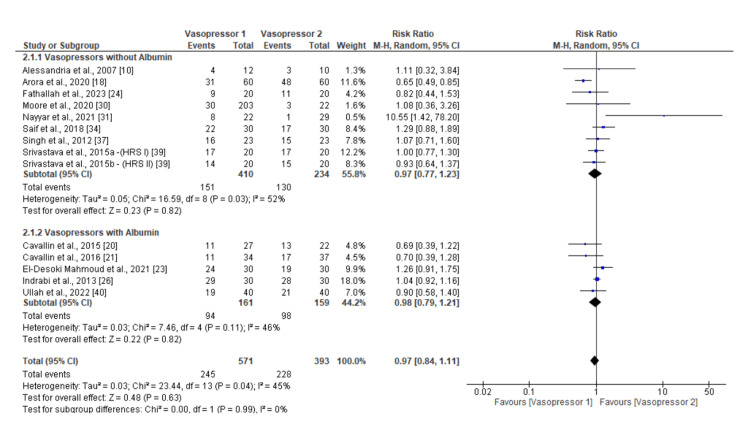
Forest plot of subgroup meta-analysis results calculating the relative morality risk of vasoconstrictors compared to each other.

Vasoconstrictors vs. vasoconstrictors: A lot of insight can be drawn from comparing the efficacy of two vasoconstrictors. In this part of the meta-analysis, a subgroup analysis was conducted to separate vasoconstrictor treatments without albumin from those with albumin. Analysis stratified to the two groups shows that neither subgroup reflects a significant difference in the rate of mortality, with RR of 0.97 (0.77 to 1.23) at 95% CI for vasoconstrictors without albumin and 0.98 (0.79 to 1.21) at 95% CI for those with albumin. The between-group heterogeneity was moderate, with I² of 52% and 46%, indicating some variability in study outcomes. Among the studies administering vasoconstrictors without albumin, the variability is statistically significant (P = 0.03). The overall effect (0.97 (0.84, 1.11)) was also not statistically significant. Figure 6 offers more detailed findings of this outcome analysis in a forest plot.

**Figure 7:**
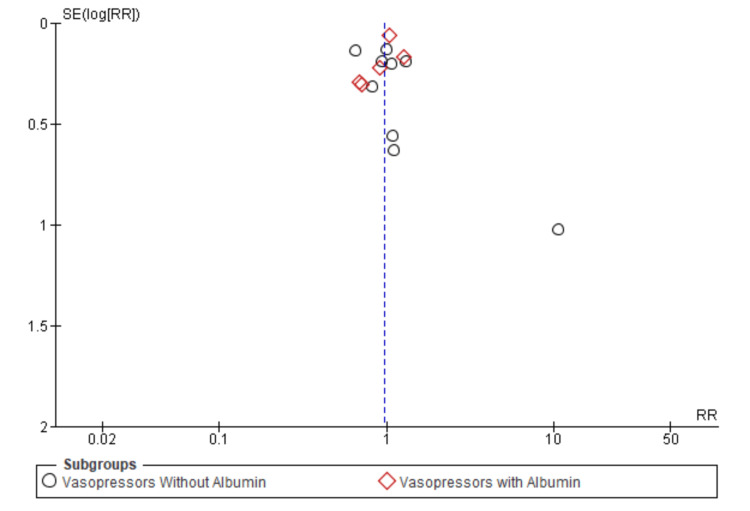
Funnel plot showing the level of publication bias between studies analyzing mortality rates. RR: risk ratio

Figure 7, which features a funnel plot, suggests a symmetrical distribution around the line of no effect (relative risk = 1), indicating minimal publication bias. However, some studies, especially those involving albumin, cluster towards the right, which may indicate small-study effects or heterogeneity within these subgroups. This symmetry is crucial for confirming the reliability of the meta-analysis results.

**Figure 8:**
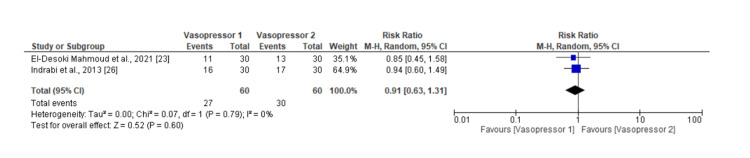
Forest plot of meta-analysis results calculating rates of HRS reversal of vasoconstrictors compared to each other. HRS: Hepatorenal syndrome

Only two studies reported data on HRS reversal comparing two vasoconstrictors. Both vasoconstrictors did not yield significantly different reversal rates. The pooled RR is 0.91, with a very wide 95% CI from 0.63 to 1.31. This indicates the level of uncertainty around the effect estimates. Low heterogeneity among the studies (I² = 0%) and a non-significant p-value for heterogeneity (P = 0.79) indicate similar results between the studies despite their methodological differences. These results are represented in Figure 8, which features a forest plot.

**Figure 9:**
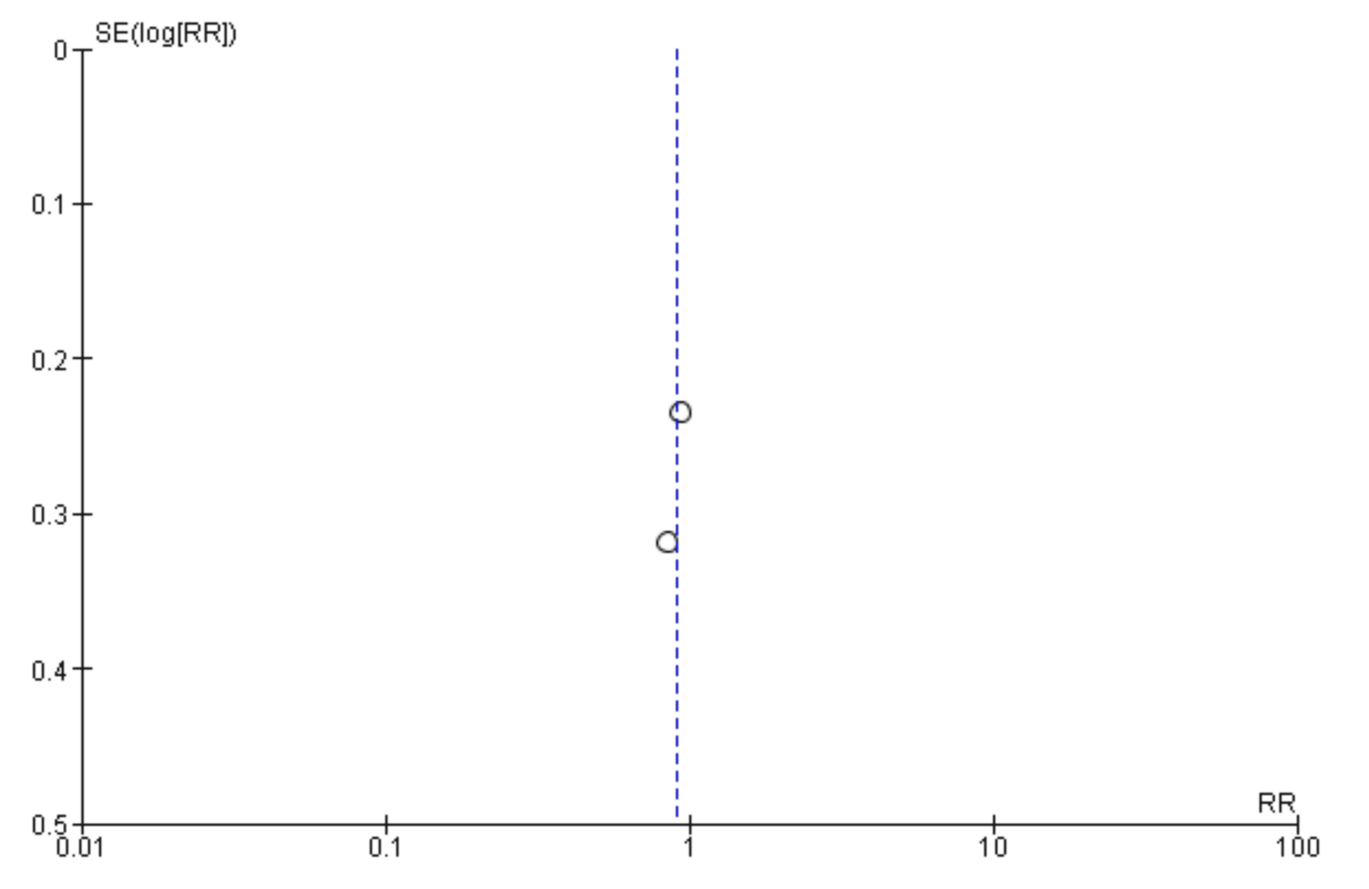
Funnel plot showing the level of publication bias between studies analyzing the rates of HRS reversal. HRS: Hepatorenal syndrome; RR: risk ratio

The funnel plot Figure 9, on the other hand, shows a symmetrical distribution of these studies, suggesting little to no publication bias between the two studies.

The meta-analysis findings have indicated that vasoconstrictors do not reduce the rate of mortality to a significant degree compared to placebo. However, there is a strong indication that the role of vasoconstrictors (especially terlipressin) in managing HRS cannot be discounted. Some of the included studies indicate a noteworthy advantage in individual subjects and raise the possibility of terlipressin effectiveness in some subpopulations or certain conditions. However, besides not being significantly effective at lowering mortality, the findings demonstrate that vasoconstrictors are twice as effective at reversing HRS. Compared to a placebo, these results dramatically increase the chance of treatment success and strongly support the use of terlipressin as an effective treatment for reversing HRS in patients. Given its proven effectiveness, clinicians may consider incorporating terlipressin as a crucial element in HRS management protocols. The mortality outcome does not vary substantially when pitting one or more vasoconstrictors for their effectiveness. The findings show that, regardless of albumin use, there is no discernible difference between the two vasopressors' efficacy in lowering mortality in HRS. Similarly, the findings imply that there is no discernible difference between the two vasopressors' efficacy in reversing HRS. This implies that other considerations, including side effect profiles, cost, and each patient's response, may be taken into account when choosing between these two vasopressors. Although this equivalency in efficacy permits clinical decision-making to be flexible, it also necessitates a cautious approach that weighs efficacy against other aspects of treatment.

Discussions

The use of vasoconstrictors, like terlipressin, has been investigated by a comprehensive body of clinical literature since its first therapeutic application for HRS in 1992. According to Aschenbrenner [45], terlipressin is now approved for clinical use in the treatment of HRS, following key recommendations from the TIPS-1 (terlipressin in patients with type 1 HRS) and TREND (terlipressin for renal function in patients with cirrhosis and complications) trials [35,46]. Significant contributions from Wong et al. [43] document its efficacy in improving renal function and HRS reversal compared to placebo. Consequently, these are markers for lowering mortality rates in HRS patients. This finding is further highlighted in the Valez et al. [41] study, which records the drug's potentiality in reducing the need for RRT. The latter is a vital measure given the significant morbidity (bleeding complications, intradialytic hypotension, and reduced long-term survival) and mortality associated with RRT in HRS patients [41,47]. In the general population (36.9% vs. 28.5%, p=0.030) and among patients who underwent liver transplantation (60.0% vs. 39.7%, p=0.010), a greater number of patients in the terlipressin group were alive and RRT-free by day 90 [41]. However, Nayyar et al. [31] and Moore et al. [30] present a more in-depth mortality analysis that indicates that, while terlipressin has overall smaller mortality rates, the difference is not necessarily statistically significant when controlled for the severity of the disease and the baseline characteristics of the patients [30]. This collection of studies highlights the complex advantages and drawbacks of employing vasoconstrictors to treat advanced cirrhosis.

Nevertheless, there are safety concerns associated with terlipressin's efficacy. Numerous studies reported significant side effects, including severe respiratory problems, vascular skin disorders, and gastrointestinal disorders which calls for careful monitoring and sparing usage of this medication. This worry has been echoed by Boyer et al. [19], stating each group saw the same amount of adverse events, although the terlipressin group's patients experienced more intestinal and other ischemia episodes. This has been reiterated by Sanyal et al. [35] and Sanyal et al. [36], who found similar rates of adverse effects. Other studies comparing terlipressin to placebo emphasize that although terlipressin can save lives, its safety profile needs to be carefully considered. Karvellas et al. [27] noted a 14% versus 5% respiratory failure rate in terlipressin and placebo groups, respectively. Similar findings were recorded by Wong et al. [43] and Wong et al. [44] stating that the incidence of respiratory failure was significantly higher in the terlipressin group.

Exploring alternative vasoconstrictor agents has led studies to investigate noradrenaline and other less commonly used types such as midodrine and octreotide. Key studies such as those conducted by Alessandria et al. [10] and Nayyar et al. [31] emphasize the efficacy of noradrenaline compared to terlipressin. While Alessandria et al. [10] found similar efficacy between the two medications, Nayyar et al. [31] demonstrated that terlipressin was more effective than noradrenaline. However, noradrenaline remains an affordable alternative. The cost comparison has also been highlighted by Saif et al. [34] and Srivastava et al. [39] who pointed out the practicality of noradrenaline particularly in situations where the HRS case is not severe. Without concrete therapeutic strategies in place, combined therapies have been experimented with to assess their efficacy. For instance, the administration of albumin seems to augment the therapeutic effects of vasoconstrictors [20,37]. The use of albumin concurrently with vasoconstrictors entailed an amelioration of renal function and, therefore, better clinical outcomes. This emphasizes the role of albumin in stabilizing patients and enhancing drug efficacy [20,37].

One unanticipated result detected by Fathallah et al. [24] was the absence of significant improvements in survival following the reversal of HRS. This suggests that reversal of HRS does not always correlate with extended survival, which is contrary to previous belief [24]. The study by El-Desoki Mahmoud et al. [23] explains that this could be because of the patients' severe liver illness, which was noted in the study. Despite better kidney function, these patients may still die from other consequences of decompensated cirrhosis that are unrelated to HRS-AKI. The study also confirmed the benefits of liver transplantation as the ultimate standard treatment for HRS and underlined its benefits [23].

This systematic review and meta-analysis offer valuable insights into the evolving landscape of clinical management strategies. By comparing these findings with the existing body of literature, my findings align with the current evidence while diverging from others. Similar to Malik et al. [48], our review confirms the efficacy of terlipressin combined with albumin in improving renal function and reducing mortality compared to noradrenaline with albumin, though with a smaller effect size for mortality reduction. This discrepancy suggests that while the benefits of terlipressin are consistent, the extent of its impact on mortality may vary depending on additional factors not fully explored in previous studies. In a similar fashion, these findings indicate a more modest mortality benefit from terlipressin than reported by Wang et al. [49], who noted a substantial reduction in mortality. This variation highlights potential differences in study populations, methodologies, or additional interventions accompanying vasoconstrictor therapy that may influence outcomes. At the same time, however, the meta-analysis found significant efficacy in HRS reversal and a reduction in mortality with terlipressin which is consistent with Gifford et al. [50]. This consistency reinforces terlipressin's role as a potent therapeutic option in HRS management, underscoring its reliability in reversing the syndrome. Echoing the moderate heterogeneity identified in the review by Israelsen et al. [51], this analysis also shows variability across studies. This is important for clinicians to consider, as it emphasizes the need to tailor HRS treatments to individual patient characteristics and conditions rather than applying a general approach. This point is consistently emphasized in this review, which is in line with the findings by Pitre et al. [52]. The study reports significant subgroup effects which resonate with this review's indication of varying degrees of response based on patient subgroups. This similarity further supports the argument for personalized medicine approaches in managing HRS [52].

Findings from other studies, seem to diverge from the results of this analysis. Contrary to Mohamed et al. [53], who observed improved kidney function without a corresponding survival benefit, our review suggests a modest survival benefit. While an individual study such as the trial by El-Desoki Mahmoud et al. [23] might reflect the opposite, the analysis still indicates possible long-term advantages of vasoconstrictor therapy that may not be immediately apparent.

This analysis enhances the current understanding of HRS management by offering a detailed analysis of vasoconstrictor effectiveness. More specifically, comparing the benefits of terlipressin and noradrenaline adds to the current understanding of these modalities. The results of this meta-analysis contribute that there is a modest yet consistent mortality benefit and significant efficacy in reversing HRS. This assertion supports the ongoing use of terlipressin, as approved by major regulatory authorities while highlighting noradrenaline as a cost-effective option, particularly in resource-limited settings. These findings emphasize the need for personalized treatment strategies, considering the varying response rates and potential side effects of these therapies. This contributes to the existing literature by promoting more nuanced clinical decision-making. Additionally, it underscores the importance of tailoring HRS treatments to individual patient profiles for optimal outcomes.

Strengths and Limitations

This analysis is based on a thorough and comprehensive methodology. One of the main strengths is its exhaustive search strategy across multiple databases, supplemented by grey literature. This approach ensured the inclusion of a wide range of studies, enhancing the breadth of the research. Secondly, the use of PRISMA guidelines provided a structured and transparent framework, facilitating the reproducibility and reliability of the findings. Additionally, the application of a random-effects model in the statistical analysis to account for clinical and methodological variability among the studies adds to the robustness of the conclusions drawn. This offers a more generalized estimate of the effects of vasoconstrictor agents on mortality rates in HRS patients.

Despite the study's comprehensive nature, several limitations may affect the reliability and generalizability of the findings. The heterogeneity among the included studies ranges between 0% and 45% which arises from variations in study populations, interventions, and outcomes measured. This element complicates data synthesis and may weaken the conclusions regarding the effectiveness of specific vasoconstrictors. Moreover, publication bias remains a critical limitation. Although funnel plots were used to assess this bias, and results indicated minimal bias, the potential underrepresentation of smaller or negative studies cannot be entirely ruled out. This may lead to an overestimation of the benefits of vasoconstrictors in treating HRS. Another notable limitation is the limited data on specific outcomes beyond mortality, such as quality of life or long-term renal function recovery. Such outcomes are crucial for fully understanding the benefits and risks associated with these treatments. These limitations suggest that while the findings provide valuable insights into the effectiveness of vasoconstrictors, they should be interpreted with caution. More importantly, due to the variability in the study populations, generalizing should be tamed when dealing with different patient populations or clinical settings. The potential for publication bias, though minimal, underscores the need for ongoing research and reporting transparency.

## Conclusions

This systematic review and meta-analysis have synthesized the current evidence on the comparative mortality rates of vasoconstrictor agents in the management of HRS. The findings indicate that while vasoconstrictors, particularly terlipressin, are effective in reversing HRS, their impact on reducing mortality is not statistically significant when compared to placebos. However, the potential of these agents to improve renal function and reduce the need for RRT is significant, underscoring their value in clinical settings. The importance of these findings lies in their contribution to refining the management of HRS, highlighting the need for tailored therapeutic strategies that consider individual patient characteristics and clinical conditions. This research supports the ongoing use of terlipressin as a critical component of HRS management protocols while also pointing to the necessity of exploring alternative treatments that could potentially offer better outcomes with fewer side effects.

Despite significant advances in the pharmacological management of HRS with vasoconstrictors, this review highlights several gaps that should be addressed in future research. First, there is a critical need for studies focusing on the long-term outcomes and quality of life of patients treated with vasoconstrictors for HRS. Research should extend beyond mortality rates to include the impact of these treatments on patient functionality, recovery duration, and overall well-being. Additionally, comparative studies between different vasoconstrictors and combination therapies could provide deeper insights into the most effective treatment protocols for various subgroups of patients with HRS. It would be valuable to explore the efficacy of newer therapeutic approaches, possibly including non-pharmacological interventions that could complement or enhance the effectiveness of vasoconstrictors. Furthermore, more rigorous RCTs with larger sample sizes and a multi-national scope are required to confirm the findings and enhance the generalizability of the results. These studies should aim to standardize intervention protocols and outcome measures to reduce variability and improve the reliability of the findings.
